# A rare presentation of large bowel obstruction post haemorrhoidectomy: a case report

**DOI:** 10.1186/s13256-023-04125-3

**Published:** 2023-09-17

**Authors:** Assia Comella, Emily Mogridge, Asiri Arachchi

**Affiliations:** Department of Surgery, Monash Health, 246 Clayton Rd., Clayton, VIC 3168 Australia

**Keywords:** Hemorrhoidectomy, Large bowel obstruction, Urinary retention

## Abstract

**Background:**

Hemorrhoidal disease is a common anorectal pathology. Complications post hemorrhoidectomy are rare. Postoperative complications following hemorrhoidectomy include bleeding (2%), infection (0.4–8%), urinary retention (15%), and constipation (15–30%).

**Case presentation:**

A 40-year-old of Asian background female presented to a tertiary colorectal service with large bowel obstruction post hemorrhoidectomy. This is the first case in the surgical literature describing large bowel obstruction secondary to extrinsic compression from urinary retention following hemorrhoidectomy. The patient developed urinary retention and obstructed defecation in setting of inadequate analgesia post hemorrhoidectomy. The patient required indwelling catheter insertion and aggressive constipation management to resolve symptoms. Histopathology from the hemorrhoidectomy did not reveal a malignancy.

**Conclusion:**

Anesthetic choice and postoperative analgesia are important factors to avoid the development of complications. A missed malignancy diagnosis must always be excluded with patients presenting post hemorroidectomy with bowel obstruction.

## Introduction

Hemorrhoidal disease is a common anorectal pathology affecting over a third of the adult population [1]. Major complications following hemorrhoidectomy are uncommon [2]. Large bowel obstruction following hemorrhoidectomy is rare, and its development can be multifactorial. It is crucial to exclude obstruction due to an undiagnosed neoplasm. The case report aims to:

(A) Ensure adequate analgesia post hemorrhoidectomy to reduce the risk of postoperative complications including urinary retention and fecal impaction
(B) Consider sinister pathology in atypical presentations of large bowel obstruction post hemorrhoidectomy (i.e., urinary/urethral or colonic/rectal/anorectal malignancy)

## Case report

We present an interesting case of a 40-year-old of Asian background female with large bowel obstruction 8 days post hemorrhoidectomy performed under general anesthesia. She presented with 3 days of abdominal pain and distension, no postoperative passage of stool or flatus, and nonbilious vomiting. Her general practitioner had started aperients without effect.

**Fig. 1:**
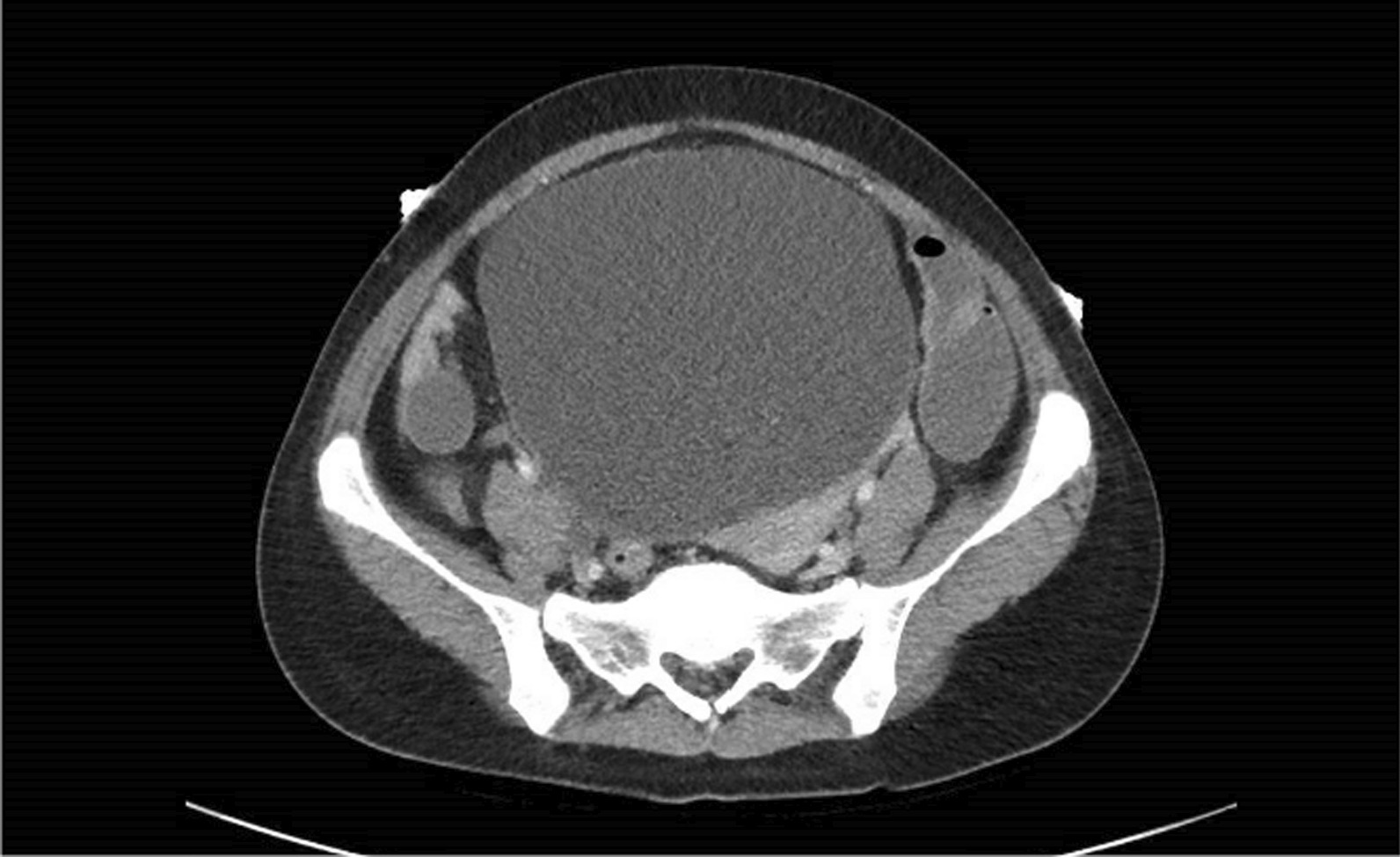
Patient’s abdomen/pelvis Computed Tomography (CT) with contrast showing urinary retention (axial view)

**Fig. 2:**
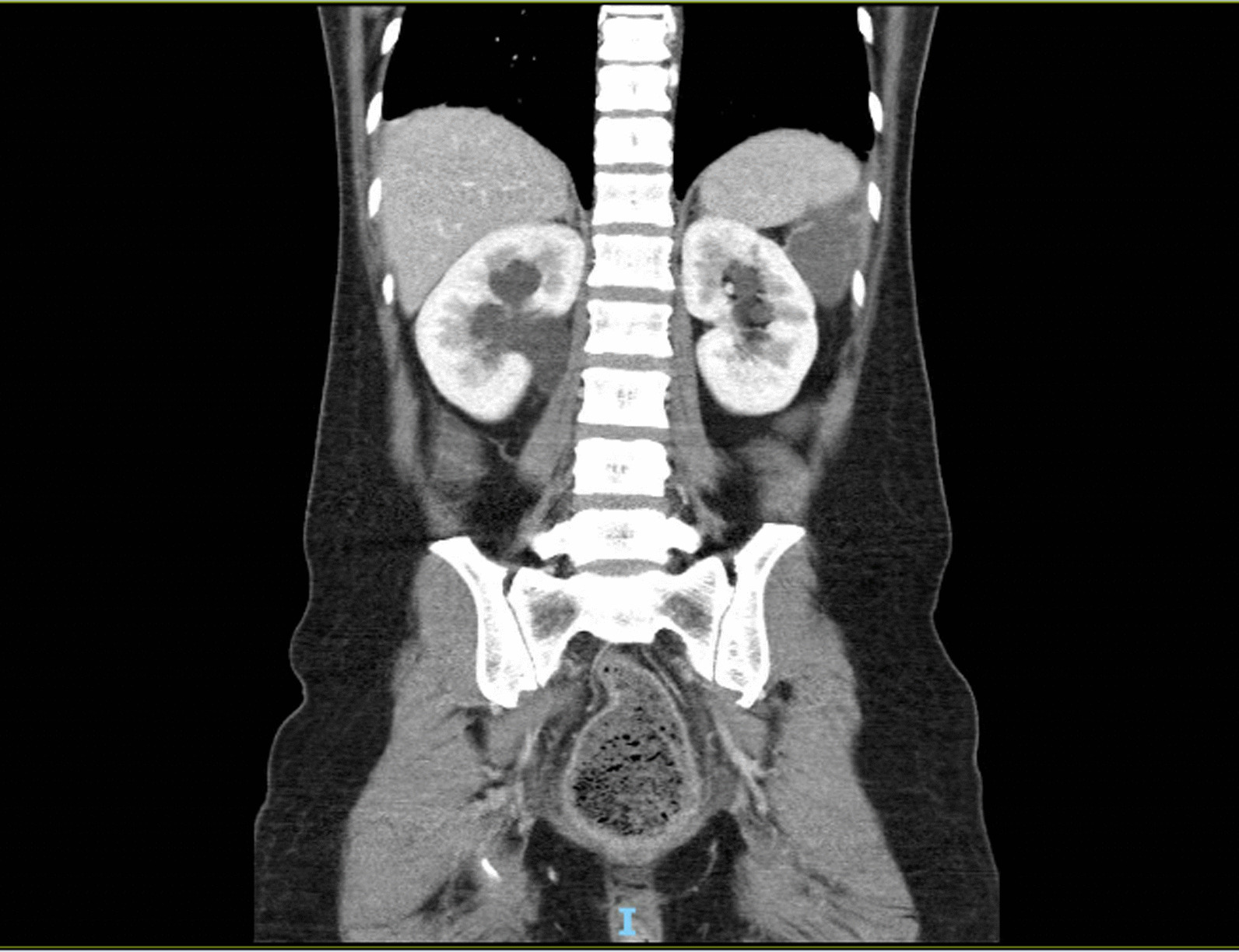
Patient’s abdomen/pelvis Computed Tomography (CT) with contrast showing hydronephrosis secondary to urinary retention (coronal view)

**Fig. 3:**
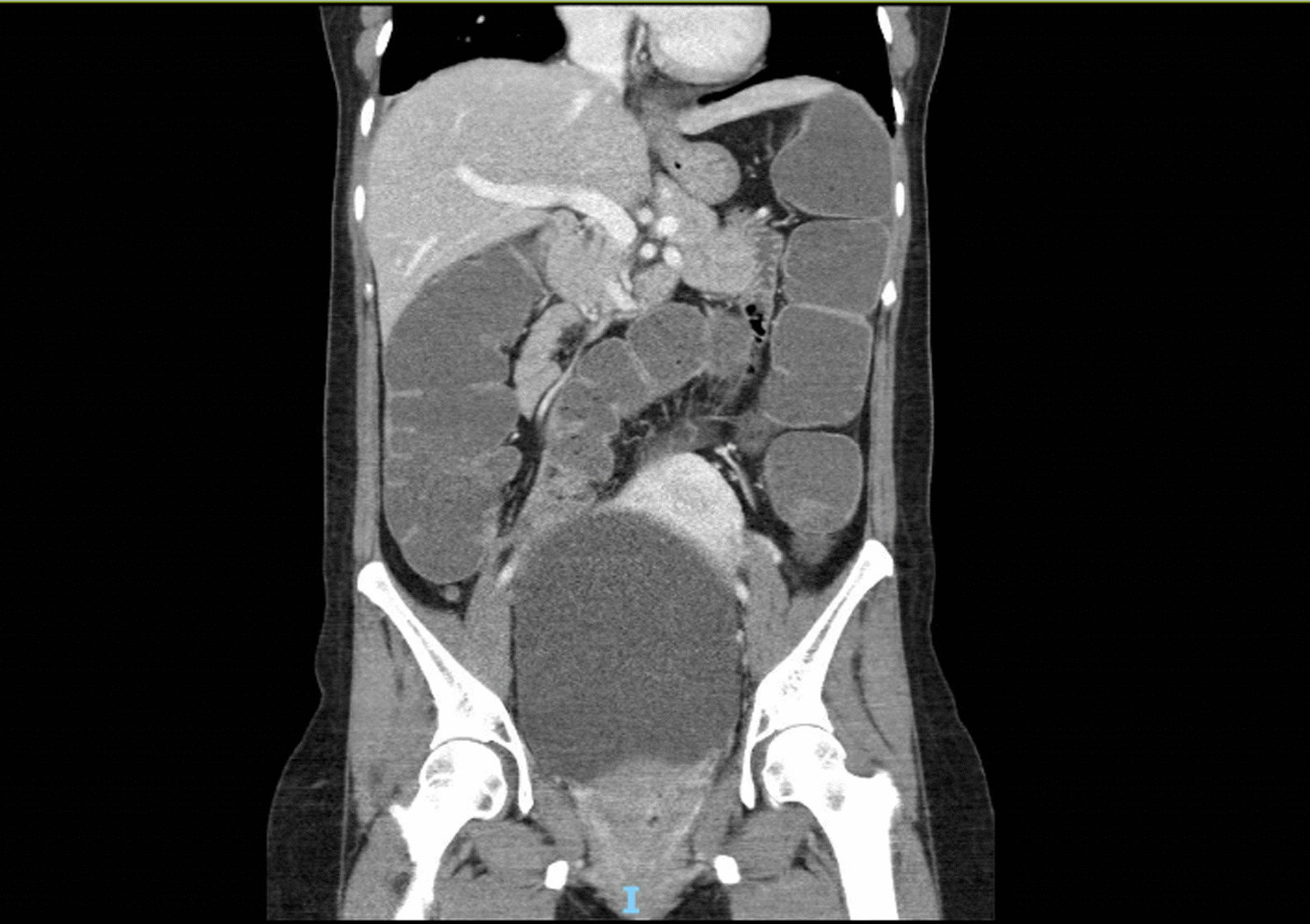
Patient’s abdomen/pelvis Computed Tomography (CT) with contrast showing dilated large bowel with transition point within the pelvis in the distal sigmoid colon, likely secondary to extrinsic compression from the distended bladder resulting in mechanical large bowel obstruction (coronal view)

Her abdomen was distended, silent, and tender across the lower abdomen without peritonism. Rectal examination revealed an external hemorrhoid, no palpable masses, no bleeding, and an empty rectum. An abdomen/pelvis computed tomography (CT) showed extremely distended bladder with bilateral hydronephrosis and hydroureter (Figs. 1, 2), and dilated large bowel with transition point within the pelvis at the distal sigmoid colon. Extrinsic compression from the distended bladder caused mechanical large bowel obstruction (Fig. 3). No sinister or malignant lesion was identified on imaging, and the patient did not report any hematuria, dyspareunia, or rectal bleeding.

An indwelling catheter (IDC) was inserted with 2.1 L urine drained, and an aggressive regimen of aperients was commenced. Stool was passed on day 1 of admission, but on day 2 trial of void (TOV) was unsuccessful. She continued on regular aperients, paracetamol, and oxycodone/naloxone 5/2.5 mg. A repeat TOV was successful on day 4. She continued aperients on discharge. Histopathology collected intraoperatively demonstrated hemorrhoids and nonmalignant fibroepithelial polyp.

## Discussion

Large bowel obstruction is a rare complication post hemorrhoidectomy, and the presented case is the first reported in literature of large bowel obstruction post hemorrhoidectomy secondary to bladder distension.

Complications post hemorrhoidectomy include bleeding (2%), infection (0.4–8%), urinary retention (15%), and constipation (15–30%) [2]. The cause of postoperative urinary retention is hypothesized to be multifactorial. A retrospective case–control study identified three independent risk factors: spinal anesthesia (odds ratio 4.41), limitation of activity (odds ratio 6.47), and pain score (odds ratio 1.80) for urinary retention post anorectal surgery [3]. Hemorrhoidectomy under general anesthesia is an independent factor for reduced urinary retention incidence (*p* = 0.044; odds ratio 2.43; 95% confidence interval 1.02–5.97) [4]. Similar to anesthetic modality, irritation/blockade of pelvic nerves during anorectal surgery is considered a contributing factor for postoperative urinary retention [2]. Perioperative fluid restriction and prophylactic analgesia significantly reduced the incidence of postoperative urinary retention [5, 6].

Similar reports in the literature of large bowel obstruction due to extrinsic compression post hemorrhoidectomy include that from Vasudevan *et al.* [7] reporting a case of a 28-year-old male who developed large bowel obstruction secondary to a large submucosal hematoma occluding the rectal lumen post stapled hemorrhoidopexy. Gallo *et al.* [8] reported a case of large bowel obstruction secondary to an extramucosal hematoma causing extrinsic compression and rectal stenosis in a 35-year-old male who underwent a hemorrhoid laser procedure.

Inadequate pain management can lead to obstructed defecation and urinary retention [9, 10]. Balancing the use of opioids to optimize analgesia while not inducing constipation and urinary retention is important [2]. Both systemic and topical agents can optimize analgesic effect. The use of topical baclofen 5% in a randomized double-blinded placebo-controlled clinical trial found a statistically significant difference, with the intervention group having reduced self-reported pain at week 1 and 2 postoperatively and using less oral analgesic medication [11].

While major complications post anorectal surgery are uncommon, this case study highlights the importance of identifying risk factors contributing to their development in order to reduce their likelihood in similar future clinical presentations and thus minimize patient morbidity [2].

As bowel obstruction is a rare complication post hemorrhoidectomy, the findings warrant the need to exclude sinister pathologies. Screening questions for malignancy symptoms and baseline investigations to exclude an obstructive malignancy (that is, urethral/cystic/colonic) should be performed. Obstructive symptoms with clinical suspicion of sinister pathology warrants exploration under anesthesia and flexible sigmoidoscopy to investigate for anorectal malignant pathology or an alternative missed sinister diagnosis.

## Conclusion

This case report depicts an interesting and rare complication post hemorrhoidectomy. Optimizing analgesia is essential in the postoperative period to limit development of urinary retention and prolonged or re-hospitalization. Avoiding spinal anesthesia in favor of general anesthesia and judicious use of intravenous fluids where able is also recommended. Considering the rarity of large bowel obstruction post hemorrhoidectomy, it is crucial to always exclude an obstructive malignancy.

## Data Availability

Not applicable.
